# Admission criteria and academic performance in first-year medical and dental students at a new medical school in Zambia: a historical cohort study

**DOI:** 10.1186/s12909-026-10171-4

**Published:** 2026-08-18

**Authors:** Kasonde Bowa

**Affiliations:** Eden University, Lusaka, Zambia

**Keywords:** Medical education, Admission criteria, Academic performance, Zambia, Sub-Saharan Africa, Health workforce, Dental education, Widening access, Cohort study

## Abstract

**Background:**

Sub-Saharan Africa carries 25% of the global burden of disease yet has only 3% of the world’s health workers. Zambia, with a population of approximately 19.7 million (2024), reported a physician density of 0.93 per 10,000 population — far below the WHO-recommended threshold. The Copperbelt University School of Medicine (CBU SOM), established in 2011, uniquely admitted two groups of first-year students: those with conventional university-level science qualifications (University group) and those with college-level dental science diplomas (College group). This created a natural experiment to examine whether alternative admission criteria compromise academic performance.

**Methods:**

A historical cohort study was conducted among all 70 first-year students admitted to CBU SOM in the academic year 2011/12. Students were grouped by admission pathway: University (*n* = 44) and College (*n* = 26). Academic performance was assessed by summated mean percentage scores across seven courses over three terms and final examinations. Primary analysis used independent samples t-tests to compare overall end-of-year performance. Secondary analyses examined performance by term, by course type, and by sex and socioeconomic subgroups. Multivariate linear regression assessed independent predictors of performance.

**Results:**

Sixty-seven students completed the full academic year. No statistically significant difference was found in overall end-of-year performance (University: 64.8% vs. College: 62.1%; *p* = 0.455). The University group performed better in Term 1 (65.3% vs. 61.2%; *p* = 0.003), Term 3 (64.0% vs. 60.0%; *p* = 0.026), and in both basic science (63% vs. 61%; *p* = 0.019) and clinical science courses (68% vs. 65%; *p* = 0.035). Female-subgroup analysis showed borderline overall difference (66.8% vs. 62.0%; *p* = 0.044) with convergence by Term 3. Socioeconomic status was the only significant predictor in multivariate analysis (*p* = 0.017); sex and age were non-significant.

**Conclusions:**

Students admitted through an alternative diploma-level pathway performed equivalently to conventionally admitted students in overall first-year academic outcomes. The University group showed an early advantage, particularly in basic sciences, which attenuated across the year — consistent with academic adaptation. Socioeconomic status was the dominant predictor of performance. These findings support the feasibility of alternative admission pathways as part of health workforce expansion strategies in low-resource settings.

This study is based on a dissertation submitted for the degree of Master of Public Health at the London School of Hygiene and Tropical Medicine, University of London, 2013 [1].

## Background

The shortage of health workers in Sub-Saharan Africa represents one of the most pressing constraints on health system performance and population health outcomes [2, 3]. The region carries 25% of the global burden of disease while having only 3% of the world’s health workers [4]. Despite the total health workforce in the WHO African Region growing from 1.98 million in 2018 to 2.67 million in 2024, critical shortages persist — particularly in physician density — and 15 countries collectively lost nearly 200,000 nurses and 3,500 doctors within just two years due to retirement and emigration [5]. A 2024 needs-based modelling study projected that the WHO Africa Region will face a health workforce shortage of more than 6 million by 2030 [6].

Zambia, with a population of approximately 19.7 million (2024 estimate), reported a physician density of 0.93 per 10,000 population — well below the WHO-recommended minimum — and remains on the WHO Health Workforce Support and Safeguards List of countries with critical shortages [7–9]. High physician attrition driven by emigration further compounds the structural deficit. For nearly half a century, Zambia relied on a single government medical school — the University of Zambia School of Medicine (UNZA SOM), established in 1966 — which graduated an average of 40 doctors per year. In 2011, the government opened a second public medical school, the Copperbelt University School of Medicine (CBU SOM), at Ndola Central Hospital. CBU SOM was notably the first institution in Zambia to offer both MBCHB and BDS programmes simultaneously [10].

The dental training pathway introduced a structural peculiarity of direct relevance to medical education research. Alongside conventional students who had completed two years of university-level natural sciences with a minimum B-grade average (the University group), CBU SOM admitted a cohort from the Dental Training College in Lusaka who held three-year diplomas in dental sciences (the College group). These diploma-entry students had not studied mathematics, chemistry, biology or physics at university level. This arrangement arose from a Ministry of Health directive to create a bridging pathway for diploma-qualified dental assistants to upgrade to full dentistry qualifications [10].

Both groups studied an identical curriculum in the first two years — seven courses spanning basic medical and clinical sciences — before separating into discipline-specific clinical training. This represented a rare natural experiment: two groups with meaningfully different academic entry qualifications undertaking the same programme in the same institution at the same time.

The question of which admission criteria best predict academic performance in medical school has attracted substantial global research. Cognitive factors — particularly prior academic achievement — explain up to 23% of variance in medical school performance [11–13]. Higher science grades and university-level pre-medical attainment predict performance in basic science examinations and likelihood of completing training [14, 15]. A 2023 longitudinal cohort study at the University of Jordan found that admission pathway and high school background significantly predicted graduation rates and GPA, with underprivileged and open-competition tracks performing comparably to conventional applicants [16]. Studies from Thailand and New Zealand have demonstrated that students admitted through equity or quota pathways perform comparably to merit-entry students by year-end, even where initial differences exist [17, 18].

Non-cognitive factors, including sex, learning style and socioeconomic background, contribute additional but smaller variance [11, 19]. Female students consistently outperform male counterparts, though this finding is most robust in larger samples. Social and demographic factors — particularly socioeconomic status — moderate the relationship between prior attainment and performance [11, 20]. Most evidence derives from high-income countries; there remains a paucity of data from African medical schools [21]. The CBU SOM cohort offered a timely opportunity to address this gap. This paper reports a historical cohort study comparing academic performance of University-entry and College-entry students over the first year of training at CBU SOM.

## Methods

### Study design and setting

A historical cohort study was conducted at the Copperbelt University School of Medicine, Ndola, Zambia, using routinely collected administrative data from the academic year 2011/12 — the inaugural cohort of CBU SOM. This paper is derived from an MPH dissertation submitted to the London School of Hygiene and Tropical Medicine in 2013 [1].

### Study population

All 70 first-year medical and dental students admitted in 2011/12 were eligible for inclusion. Three students who did not complete the full academic year were excluded from analysis, leaving 67 students: 43 in the University group and 24 in the College group.

### Group definitions

The University group comprised students admitted on the basis of university-level training in natural sciences (BSc Natural Sciences or Health Sciences), with a minimum average grade of B (65–74%) across four science courses. The College group comprised students admitted on the basis of a three-year Diploma in Dental Sciences from the Dental Training College, Lusaka, with a minimum average B grade in final-year dental science courses.

### Outcome measures

The primary outcome was the summated mean percentage score across all seven first-year courses at end of year. Secondary outcomes were: (i) mean scores per term (Terms 1, 2, and 3); (ii) mean scores in basic science courses (five courses) versus clinical science courses (two courses); and (iii) mean scores in female-only and low-socioeconomic-status female subgroups.

### Covariates

Variables collected from student records included: admission group (University or College), age, sex, training programme (medicine or dentistry), and socioeconomic status. Socioeconomic status was operationalised using the Ministry of Education bursary classification: low (100% government funding), middle (partial funding), or high (no government funding required).

### Statistical analysis

Data were extracted onto anonymised capture sheets and entered into Microsoft Excel by two independent clerks. Analysis was performed using STATA version 8.0. Independent samples t-tests compared mean scores between groups (significance: *p* < 0.05). Secondary analyses used sub-stratification by sex and socioeconomic status to control for confounding. Multivariate linear regression modelled the contribution of age, sex, and socioeconomic status to overall performance.

### Ethics approval and consent to participate

Ethics approval was obtained from the Tropical Diseases Research Centre (TDRC) Ethics Committee, Ndola, Zambia, and from the London School of Hygiene and Tropical Medicine Research Ethics Committee, London, UK. All examination data were fully anonymised prior to analysis. No individual student was identifiable from the data. As this study used only routinely collected, anonymised administrative records and involved no direct contact with students, a waiver of individual consent was approved by both committees. All research was conducted in accordance with the Declaration of Helsinki.

## Results

### Study population characteristics

Sixty-seven students were included in the final analysis. Table 1 summarises baseline characteristics. The two groups differed significantly in mean age (University: 27.6 years; College: 32.6 years; *p* = 0.0005), with the College group on average five years older. Male students predominated in the University group (male: female ratio 1:0.54) while the College group had an approximately equal sex distribution (1:1.00), though this difference did not reach statistical significance (*p* = 0.16). The groups also differed significantly in socioeconomic status distribution (*p* = 0.004), with the College group more concentrated in the middle income category.

**Table 1:** Baseline characteristics of study participants by admission group

Characteristic	Group 1 (University) *n* = 43	Group 2 (College) *n* = 24	*P* value	95% CI
Male : Female ratio	28:15 (1:0.54)	12:12 (1:1.00)	0.16	—
Mean age (years)	27.6	32.6	0.0005	2.3–7.7
Age range (years)	23–43	21–43	—	—
SES: Low / Middle / High	31.8% / 38.6% / 29.6%	23.1% / 61.5% / 15.4%	0.004	—

Three students who withdrew before completing the academic year were excluded from analysis

*SES* socioeconomic status

*p < 0.05 considered statistically significant

### Primary analysis: overall academic performance

No statistically significant difference was observed in overall end-of-year performance between the University group (64.8%) and the College group (62.1%) (*p* = 0.455; 95% CI: −10.1 to 4.6) (Table 2). However, the University group performed significantly better in Term 1 (65.3% vs. 61.2%; *p* = 0.003) and Term 3 (64.0% vs. 60.0%; *p* = 0.026). Both groups showed a decline in mean marks in Term 2, recovering in Term 3. The Term 2 difference was not statistically significant (57.5% vs. 54.9%; *p* = 0.124). When courses were grouped by discipline, the University group performed significantly better in both basic science (63% vs. 61%; *p* = 0.019) and clinical science courses (68% vs. 65%; *p* = 0.035).

**Table 2:** Primary analysis: academic performance by term and course type

Assessment	Group 1 (University) Mean %	Group 2 (College) Mean %	*P* value	95% CI
Overall end-of-year result	64.8	62.1	0.455	−10.1 to 4.6
Term 1 result	65.3	61.2	0.003*	1.3–6.8
Term 2 result	57.5	54.9	0.124	−0.7 to 6.0
Term 3 result	64.0	60.0	0.026*	0.5–7.5
Basic science courses	63.0	61.0	0.019*	0.3–3.7
Clinical science courses	68.0	65.0	0.035*	0.2–5.8

*CI* confidence interval

*p < 0.05

### Secondary analysis: female subgroup

Analysis restricted to female students (University *n* = 15; College *n* = 12) showed a borderline overall difference in end-of-year performance (66.8% vs. 62.0%; *p* = 0.044) (Table 3). The University group performed significantly better in Term 1 (66.2% vs. 60.9%; *p* = 0.009) and Term 2 (60.0% vs. 55.7%; *p* = 0.028). The performance gap narrowed by Term 3, where the difference was no longer statistically significant (65.0% vs. 61.0%; *p* = 0.069). The University female group also performed better in clinical sciences (71.2% vs. 66.4%; *p* = 0.046), but no significant difference was seen in basic sciences (65% vs. 61%; *p* = 0.112). The mean age difference between female subgroups was not statistically significant (27.1 vs. 30.7 years; *p* = 0.06).

**Table 3:** Secondary analysis: academic performance in female students only

Assessment (females only)	Group 1 (University) *n* = 15	Group 2 (College) *n* = 12	*P* value	95% CI
Overall end-of-year result	66.8%	62.0%	0.044*	0.1–9.5
Term 1	66.2%	60.9%	0.009*	1.5–9.1
Term 2	60.0%	55.7%	0.028*	0.5–8.1
Term 3	65.0%	61.0%	0.069	−0.3 to 8.3
Basic science courses	65.0%	61.0%	0.112	−1.0 to 9.0
Clinical science courses	71.2%	66.4%	0.046*	0.1–9.5

*p < 0.05

### Secondary analysis: socioeconomic subgroup

When analysis was further restricted to females of low socioeconomic status (University *n* = 6; College *n* = 14), no significant difference in overall end-of-year performance was observed (62% vs. 59%; *p* = 0.570). The only significant difference was in basic science courses (70% vs. 58%; *p* = 0.025). Wide confidence intervals in this analysis reflect small subgroup sizes.

### Multivariate analysis

Multivariate linear regression incorporating sex, age, and socioeconomic status as predictors of overall academic performance yielded an adjusted R² of 11.6% (Table 4). Only socioeconomic status reached statistical significance (*p* = 0.017), with a negative coefficient (− 2.52), indicating that higher socioeconomic category was associated with lower performance. Age (*p* = 0.118) and sex (*p* = 0.923) were non-significant predictors.

**Table 4:** Multivariate linear regression: predictors of overall academic performance

Variable	Coefficient	Standard Error	*P* value	95% CI
Intercept	75.01	4.73	< 0.001	65.6–84.5
Sex	−0.15	1.50	0.923	−3.1 to 2.8
Age	−0.20	0.12	0.118	−0.44 to 0.05
Socioeconomic status	−2.52	1.03	0.017*	−4.6 to − 0.5

*p < 0.05. Adjusted R² = 0.116. Overall model F(3,63) = 3.90, *p* = 0.013

## Discussion

### Principal findings

This historical cohort study found no statistically significant difference in overall first-year academic performance between students admitted through conventional university-level science training and those admitted through a diploma-level dental sciences pathway. Both groups achieved end-of-year means within two to three% points of each other. When potential confounders — age, sex, and socioeconomic status — were controlled through subgroup stratification and multivariate regression, performance differences became smaller and largely non-significant.

The most consistent finding was that the University group performed better in basic science courses — unsurprising given their university-level prior training in mathematics, chemistry, biology and physics. More striking was the attenuation of performance gaps across the academic year. The University group’s advantage was most pronounced in Term 1, diminished in Term 2, and partially re-emerged in Term 3. The female-only subgroup analysis most clearly illustrates this trajectory. This pattern is consistent with evidence from equity-pathway admissions programmes globally — including studies from New Zealand, Thailand, and Jordan — that students from non-traditional entry backgrounds narrow performance gaps with conventional-entry peers as they adapt to the academic environment [16–18].

### Socioeconomic status as the dominant predictor

The finding that socioeconomic status was the only statistically significant predictor in the multivariate model — with sex and age non-significant — has important implications. The negative coefficient for SES suggests that students from lower socioeconomic backgrounds performed at least as well as their more affluent peers. This finding aligns with evidence from resource-limited settings where government-sponsored students may carry stronger motivational drivers relative to financial investment [20, 22]. It also complicates the primary group comparison: the College group, more concentrated in the middle SES category, may have been disadvantaged by this distribution.

### The Term 1 effect and academic adaptation

The University group’s significantly better performance in Term 1 (*p* = 0.003), disappearing in Term 2 (*p* = 0.124), is an intriguing finding. A plausible explanation is that university-trained students enter with established academic study habits and familiarity with university examination formats — advantages that provide an early edge but normalise once diploma-entry students have settled in. This is consonant with evidence that study skills are teachable and performance gaps at entry do not necessarily predict long-term outcomes [17, 19]. The partial re-emergence in Term 3, when both term and year-end examinations coincide, may reflect differential coping with cumulative examination load.

### Policy implications

These findings have direct relevance to health workforce policy in Zambia and across Sub-Saharan Africa. A number of countries are pursuing strategies to accelerate health worker production by expanding the pool of eligible candidates, including through bridging programmes that enable diploma-qualified mid-level workers to upgrade to full professional qualifications [2, 3, 21]. The CBU SOM experience provides preliminary empirical support for the feasibility of such approaches. However, the finding of an early basic science disadvantage for College-entry students suggests that targeted academic support in the first term and in science disciplines could optimise outcomes for this group.

### Limitations

Several limitations must be acknowledged. The sample was small (*n* = 67), and subgroup analyses were limited by further reductions in group size, resulting in wide confidence intervals. The cohort was from a single institution in a single year, limiting generalisability. The two groups differed significantly at baseline in age, sex, and socioeconomic status, creating a confounded comparison not fully resolved by multivariate analysis. The historical cohort design precluded collection of qualitative data — including motivation, learning styles, and study habits — that may have enriched interpretation. Attendance was not captured as it was not compulsory. The one-year follow-up is short; longitudinal tracking through clinical years and professional practice would substantially strengthen the evidence base.

### Future research

Replication across subsequent CBU SOM cohorts would allow assessment of whether these preliminary findings hold as the school matures. Longitudinal follow-up — particularly through the clinical years where College-entry students’ prior practical healthcare experience might confer advantages — would be informative. Qualitative work exploring the experiences of diploma-entry students and their adaptive strategies would complement the quantitative evidence.

## Conclusions

In the inaugural cohort of a new Zambian medical school, students admitted through an alternative diploma-level pathway performed equivalently to conventionally admitted students in overall first-year academic outcomes. The University-entry group showed a consistent early advantage in Term 1 and in basic science disciplines, which attenuated across the year. Socioeconomic status — not admission pathway — was the sole significant predictor of academic performance in multivariate analysis. These findings provide empirical support for the selective use of alternative admission criteria as part of a strategy to expand health professional training capacity in Sub-Saharan Africa, subject to appropriate academic bridging support and confirmation in larger, longer-term cohorts.

## Data Availability

All data supporting the findings of this study are available within the paper.

## Abbreviations

BDS: Bachelor of Dental Surgery
BMI: Body Mass Index (not used)
CBU SOM: Copperbelt University School of Medicine
CI: Confidence Interval
GPA: Grade Point Average
HRH: Human Resources for Health
LSHTM: London School of Hygiene and Tropical Medicine
MBCHB: Bachelor of Medicine and Bachelor of Surgery
MOE: Ministry of Education
MOH: Ministry of Health
SES: Socioeconomic Status
SSA: Sub-Saharan Africa
TDRC: Tropical Diseases Research Centre
UNZA SOM: University of Zambia School of Medicine
WHO: World Health Organization
